# Testing a simplified tool and training package to improve integrated Community Case Management in Tanganyika Province, Democratic Republic of Congo: a quasi-experimental study

**DOI:** 10.7189/jogh.09.010810

**Published:** 2019-06

**Authors:** Anne Langston, Alison Wittcoff, Pascal Ngoy, Jennifer O’Keefe, Naoko Kozuki, Hannah Taylor, Yolanda Barbera Lainez, Sambou Bacary

**Affiliations:** 1 International Rescue Committee, New York, New York, USA; 2 International Rescue Committee, Kinshasa, DR Congo; 3 World Health Organization, Kinshasa, DR Congo

## Abstract

**Background:**

Integrated community case management (iCCM) is a strategy to train community health workers (*relais communautaires* or *RECOs* in French) in low-resource settings to provide treatment for uncomplicated malaria, pneumonia, and diarrhea for children 2-59 months of age. The package of Ministry of Public Health tools for *RECO*s in the Democratic Republic of Congo that was being used in 2013 included seven data collection tools and job aids which were redundant and difficult to use. As part of the WHO-supported iCCM program, the International Rescue Committee developed and evaluated a simplified set of pictorial tools and curriculum adapted for low-literate *RECO*s.

**Methods:**

The revised training curriculum and tools were tested in a quasi-experimental study, with 74 *RECO*s enrolled in the control group and 78 *RECO*s in the intervention group. Three outcomes were assessed during the study period from Sept. 2015-July 2016: 1) quality of care, measured by direct observation and reexamination; 2) workload, measured as the time required for each assessment – including documentation; and 3) costs of rolling out each package. Logistic regression was used to calculate odds ratios for correct treatment by the intervention group compared to the control group, controlling for characteristics of the *RECOs*, the child, and the catchment area.

**Results:**

Children seen by the *RECO*s in the intervention group had nearly three times higher odds of receiving correct treatment (adjusted odds ratio aOR = 2.9, 95% confidence interval CI = 1.3-6.3, *P* = 0.010). On average, the time spent by the intervention group was 10.6 minutes less (95% CI = 6.6-14.7, *P* < 0.001), representing 6.2 hours of time saved per month for a *RECO* seeing 35 children. The estimated cost savings amounts to over US$ 300 000 for a four-year program supporting 1500 *RECO*s.

**Conclusion:**

This study demonstrates that, at scale, simplified tools and a training package adapted for low-literate *RECO*s could substantially improve health outcomes for under-five children while reducing implementation costs and decreasing their workload. The training curriculum and simplified tools have been adopted nationally based on the results from this study.

While huge strides have been made globally, the under-five mortality rate still remains unacceptably high in many sub-Saharan African countries. According to the World Health Organization [1], children born in sub-Saharan Africa are 14 times more likely to die before their fifth birthday compared to children in developed regions. It is estimated that malaria, diarrhea and pneumonia account for 37% of all under-five deaths in sub-Saharan Africa [2]. To reduce under-five mortality, many low-income countries have adopted integrated community case management (iCCM), a WHO-recommended equity-focused strategy to improve access to care beyond health facilities for children 2-59 months, in which community health workers (*RECO*s) receive training and supervision to treat uncomplicated malaria, pneumonia and diarrhea in their communities and refer complicated cases [3].

Although evidence exists to show that, if well-implemented, iCCM can contribute to saving lives of children under five [4], many recent studies have also revealed the numerous bottlenecks in implementation which may prevent iCCM from having its intended benefit, including poor quality of services [5]. However, there is limited research on factors influencing the quality of care, specifically the impact of training methods, job aids and reporting tools on the quality of care and workload of *RECO*s [6,7]. Many countries have tools and training curricula that are not adapted for low-literate *RECOs*, resulting in errors during assessment of the sick child and long consultation times [8]. In the Democratic Republic of Congo (DRC), the Ministry of Public Health (MoPH) tool package for *RECOs* includes seven highly redundant reporting forms and several job aids which are difficult to use and require a high level of literacy [9,10].

From September 2013 to November 2017 the International Rescue Committee (IRC) implemented the Rapid Access Expansion (RAcE) project in Tanganyika Province with the support of the World Health Organization. Working under the leadership of the MoPH, the program trained and supported approximately 1600 *RECOs*, serving an estimated 360 000 children under five. As part of the RAcE-supported initiative, the IRC developed a curriculum and a simplified set of pictorial tools adapted for low-literate *RECOs*. The purpose of this study was to evaluate the effect of the package on quality of care, *RECO* workload and program implementation costs. Due to resource constraints and scope of work limitations, this study was unable to look at other factors that may have had an impact on quality of care such as supervision.

## METHODS

### Context and program design

The study was conducted from September 2015 to July 2016 in Tanganyika Province, DRC. Tanganyika Province, located in conflict-affected Eastern Congo, covers 150 940 km^2^ with a total population estimated at 2 649 317 and a density of 18 inhabitants per square kilometer. Access to health care services is limited by financial and geographic barriers. According to results of the 2013-2014 DRC-DHS, under five-mortality was 121 per 1000 live births and malaria prevalence was 32% among children 6-59 months in Katanga province (which at the time included Tanganyika) [11].

In an effort to address barriers to health services, DRC began implementing iCCM in 2005. As described in the MoPH implementation guide [9], Relais Communautaires, often referred to as *RECO*s in DRC, are unpaid volunteers selected by their communities. They must be literate and have a source of income, limiting the pool of candidates and excluding most women. In the RAcE-supported program, less than 10% of *RECOs* were women. *RECOs* were trained to assess and classify children 2-59 months of age presenting with signs of illness and treat uncomplicated cases of malaria, pneumonia and diarrhea, and refer children with severe illness after giving the first dose of medication. *RECOs* are supervised by the head nurse of the health center located closest to the *RECOs*’ catchment area. Additional supervision was provided by the health zone, regional authorities and IRC project staff.

### Revised tools

The MoPH package (control) consisted of seven separate tools that must be completed by the *RECOs* (**Table 1**). Due to the complexity of the tools, much of the training is dedicated to teaching *RECOs* how to use them, leaving little time for building skills in assessment and treatment. To address these concerns, the IRC in collaboration with MoPH developed a simplified tool package, henceforth referred to as the intervention. The tools were based on tools that IRC had tested and implemented in Sierra Leone and South Sudan, incorporating best practices for low-literacy communication. The total number of tools used by the *RECOs* decreased from seven to four, limiting the data collected to that used for supervision and program management (**Table 1**). Supervision guidelines, checklists and incentives were not altered by the study.

**Table 1:** Summary of tools in control and intervention packages

Name	MoPH package (control)	Revised package (intervention)
1. Individual Sick Child Form	Takes the *RECO* through steps of sick child management, documenting the findings of the assessment and decisions made. 89 data points.	Replaced with pictorial job aids which provide images and step by step instructions for assessment, classification, treatment, counseling, and referral. Laminated cards given during the training. No data collected.
2. Referral Note	Used for referral of a child with danger signs or another condition the *RECO* is not trained to treat; includes reason for referral and pre-referral treatment provided.	Includes images of danger signs and images of pre-referral treatment that the *RECO* ticks to inform the health center of the reason for referral and treatment given.
3. Register	Includes all cases assessed by the *RECO* during the month and notes whether the child had any danger signs, any procedures carried out during the assessment (malaria testing, middle upper arm circumference (MUAC) measurement, and breath count), the classification of the child, and the treatment received. 30 data points.	Similar to the original register, noting all assessment steps and classifications, but uses images which match job aids as guidance. The register also captures drug management of all medications and supplies that the *RECO* uses monthly. 32 data points.
4. Medication Count Notebook	Notes the number of each medication given out each day by the *RECO.*	Replaced by the register.
5. Medication Stock Register	Notes the medications received by the *RECO* from the health center and the medications used for treatments to children each month. 13 data points.	Replaced by the register.
6. Medication Order Form	Used for requesting medications to the head nurse at the health center each month.	No change.
7. Monthly Report	Summarizes aggregated data on sick children treated and stock management in a reporting month.	No change.

MoPH – Ministry of Public Health, *RECO – relais communautaires*

### Training package

The curriculum developed for the intervention was based on best practices of adult learning methodologies and incorporated activities such as role plays and peer discussion. The images from the job aids and registers, along with large format posters, were used throughout to help reinforce learning. Both trainings lasted six days and were guided by a curriculum which provided a detailed training agenda. Because the tools in the intervention were less complicated, the *RECOs* in the intervention group were able to spend more time on practical skills during their training.

### Study design and sampling

This was a quasi-experimental study conducted to compare the quality of care and workload between *RECOs* trained and deployed with the current MoPH package and those using the intervention package. Quality of care was evaluated using direct observation and reexamination, a method found in previous research to be the most accurate measure of *RECOs* quality of care [12]. A quality of care evaluation earlier in the program (2014) found that 44% of cases were treated correctly by the *RECOs*. We sought to detect a relative 50% improvement to the earlier assessment (a 66% correct treatment rate), with an alpha of 5% and a power of 80%. With a 10% loss-to-follow-up incorporated, we estimated 79 *RECOs* for each arm.

Among the health zones covered by the program, Kabalo and Manono were selected for the study because iCCM had not yet been scaled up to those zones. Both health zones are remote, with populations between 66 000 and 67 000, and 100 *RECOs* each. Within the zones, equal numbers of health center catchment areas were selected to participate in the operational research. Health center catchment areas were used as the unit of assignment to prevent contamination between the two groups. Health center catchment areas that had already begun training *RECOs* with the MoPH package were used as control areas, with about half of the areas in each of the respective health zones assigned to the intervention package. Within each health area, all *RECOs* received the same training and tools.

*RECOs* in the control group were trained from February to March 2015, and the intervention group was trained from July to September 2015. Trainings were staggered based on the timing of the project scale-up schedule. Both sets of trainings were preceded by a training of trainers facilitated by MoPH personnel with support from RAcE Zonal Supervisors. As per MoPH policy, head nurses from the selected health areas participated in the trainings to support the *RECOs* they would be supervising.

### Quality of care evaluations

Among those trained on either package, *RECOs* who had been active for at least six months and had submitted their monthly reports for the preceding two months were included in the evaluation sample.

The quality of care evaluations were conducted six to seven months after each of the trainings— September 2015 for the control group and March 2016 for the intervention group. Because of staff availability and time constraints, a single team performed the first evaluation, while two teams conducted the second. The supervisor from the first evaluation trained all evaluators for both teams. All evaluation teams consisted of one supervisor and two evaluators, a trained IRC clinician and the MoPH zonal focal point. For the second evaluation, a provincial focal point served as one of the evaluators. In total, 154 *RECO*s were evaluated, 75 in the control arm and 79 in the intervention arm. **Table 2** below shows the breakdown of health centers and *RECOs* assigned to each arm per zone. More *RECO*s were trained than evaluated because some *RECOs* did not complete the initial training, were deemed incapable of providing services at the end of the training, or had resigned within the first six months. In addition, our sample size calculations did not require us to evaluate all *RECO*s who had been trained.

**Table 2:** Health centers participating in operational research per arm by zone

	Control			Intervention		
**Health yone**	**No. of health centers**	**No. of *RECOs* trained**	**No. of *RECOs* observed**	**No. of health centers**	**No. of *RECOs* trained**	**No. of *RECOs* observed**
Kabalo	14	55	38	10	45	39
Manono	10	51	36	15	49	39
Total	24	106	74	25	94	78

RECO – relais communautaires

The evaluations were conducted at the health facility where the *RECOs* were asked to be present on a prearranged day. The selection of *RECOs* for evaluation was done by zonal project supervisors, and based on availability, possibly resulting in a bias in favor of higher performing *RECOs*, although this would have been equally true for both arms. Evaluations were conducted at the health facility because more time and resources would have been required if each team had to travel to the home of each *RECO* assessed. In addition, there would have been no guarantee of a sick child coming to the *RECO’s* home on the day the evaluation team was present. All *RECOs* used an identical kit containing all the necessary drugs, materials, and tools for assessment of a sick child. The children assessed during the evaluation were selected by the head nurse from among the sick children brought to the facility that day. The criteria given to the head nurse was to exclude children who were so ill that a slight delay in treatment would put them at risk.

*RECOs* were instructed to assess the child using the materials and tools provided, in the same manner as if they were providing care at their home. They were advised that they should present the necessary medications to the mother during the consultation, but not administer the first dose, as would normally be done. The evaluators observed the assessment and filled out a checklist to document the *RECO’s* findings and decisions. All the *RECOs* evaluated were asked to count breaths for the child they were assessing, regardless of the condition. The *RECO’s* count was compared to the count of the clinician and considered correct if it was within ± three breaths. Each *RECO* was observed providing case management to one sick child.

After the assessment, the *RECO* was taken to a separate area to complete filling out the rest of their tools (if necessary) and the trained clinician then reassessed and classified the child’s condition, giving treatment according to iCCM protocol. The checklists of the two data collectors were cross-examined by the evaluation team for completeness and consistency. Discrepancies were discussed and corrected immediately after the process was completed, before proceeding to the next assessment.

### Analysis

Data were entered into Excel (Microsoft Inc, Seattle WA, USA) and cleaned using Excel and Stata 11 (Stata Corp, College Station, TX, USA). Data reconciliation was done in Kalemie, DRC, and New York, USA. Unadjusted and adjusted logistic regression models were run to calculate the odds ratios of correct performance per protocol, comparing *RECOs* trained in the intervention model against the control. The outcome variables of interest are presented in **Table 3**.

**Table 3:** Quality of care outcomes

Outcome	Definition
Correct assessment of danger signs	*RECO* checked for all danger/alert signs as per national protocol, which includes: vomits everything, convulsions, unable to drink/breastfeed, blood in stool, frequently sick, treatment failure, cough for 14 d or more, diarrhea for 14 d or more, fever for 7 d or more, red MUAC, palmar pallor, edema, severe visible wasting, unconscious or lethargic, diarrhea with dehydration, chest in-drawing, fever with generalized skin rash, very weak. MUAC is only taken for children 6 mo or older.
Correct referral decision	*RECO* referred the child for a danger/alert sign that was present or if the child presented with any condition outside of the iCCM conditions.
Correct respiratory count	*RECOs* count of respiratory movements of child was within ±3 of gold standard (trained clinician) after one minute.
Correct classification of individual conditions	Diarrhea: Caregiver reported diarrhea and *RECO* classified as diarrhea.
	Malaria/fever: Caregiver reported fever, RDT was conducted, and *RECO* classified case as malaria if positive and fever if negative.
	Pneumonia/cough: Caregiver reported cough or difficulty breathing, respiratory rate was measured and *RECO* correctly classified case as pneumonia or cough.
Correct treatment of individual conditions	Diarrhea: *RECO* correctly classified diarrhea and gave correct dose of both zinc and ORS according to age
	Malaria: *RECO* correctly classified malaria or fever based on RDT and gave correct dose of ACT and/or paracetamol according to classification and age.
	Pneumonia/cough: *RECO* correctly classified pneumonia or cough and gave correct dose of amoxicillin for age or counselling on home remedies according to classification.
Correct overall management	*RECO* correctly classified all conditions, including referral, and correct treatment for age, including pre-referral treatment when indicated.

RECO – relais communautaires

The regression models controlled for potential confounders in two categories: *RECO* characteristics (age, sex, education [less than complete secondary/complete secondary or more], occupation [subsistence/professional], the health zone in which the *RECO* worked, the characteristics of the child (age, sex, condition [fever/diarrhea/respiratory symptoms], and the complexity of the child’s condition [one condition/multiple conditions/three conditions or a non-iCCM condition/any danger sign]. The analyses were performed in Stata 11 (StataCorp LP, College Station, TX). *P*-value of <0.05 was considered statistically significant.

### RECO workload

The total time the *RECO* spent on the assessment and documentation was recorded in minutes and seconds and entered into the study database. Linear regression was used to assess whether there was a difference between the two models, controlling for the same confounders listed above.

#### Cost analysis

In assessing the cost difference between the two models, we focused on areas of implementation where costs were expected to be different: printing of tools and training materials, and distribution of tools to *RECOs*. Actual costs and cost estimates from similar activities were used to create a prototype budget for one health zone with 100 active *RECOs* under each of the models. The costs for distribution of tools took into account the gas needed to cover all of the routes in Kabalo health zone, which is similar in size to other health zones in the region, covering a distance of 1706 km to reach all relevant *RECO*s.

Microsoft Excel (Microsoft Inc, Seattle, WA, USA) was used for the workload and cost analyses.

### Ethics

Verbal consent was obtained from the *RECO* and each child’s caregiver using standard consent forms. Ethical approval was obtained from the Institutional Review Boards of the Lubumbashi School of Public Health and the IRC respectively.

## RESULTS

### Characteristics of the RECOs

Of the eligible 158 *RECOs*, (79 from each arm), four from the control arm were unable to participate in the assessment due to sickness or a death in the family and were dropped. Additionally, one *RECO* from each arm was dropped because the evaluators did not apply the definition of correct treatment consistently. The two groups of *RECOs* differed significantly in age, sex, and education, as shown in **Table 4**. The intervention group was younger on average (average age 36.3, compared to 43.2 in the control group, *P* < 0.000), included fewer women (18% in the control and 10% in the intervention, *P* = 0.192), and had a higher level of education (58% has completed at least secondary school, compared to 34% in the control, *P* = 0.013). All sampled *RECOs* were supervised by the head nurse from their health area. Research personnel had no influence on head nurse work assignments, and policies and procedures were the same across the two groups. However, it is reasonable to expect that the quality of supervision provided would affect quality of care. The only supervision data available was the number of supervisions in the health area, at best a weak proxy for the quality of the supervisor’s involvement. In regression analysis, no relationship was found between the average number of supervisions in the health zone and any of the performance indicators, but it was retained in the regression as a control variable.

**Table 4:** Demographic characteristics of the *RECOs* evaluated by health zone and model

	Kabalo		Manono		Combined	
	**Control (N = 38)**	**Intervention (N = 39)**	**Control (N = 36)**	**Intervention (N = 39)**	**Control (N = 74)**	**Intervention (N = 78)**
**Age:**						
Mean	41.5	35.2	44.9	37.4	43.2	36.3*
Median (range)	44 (28-70)	33 (22-66)	41 (24-69)	34 (21-58)	43 (24-70)	33 (21-66)
**Sex:**						
Female	8 (21%)	1 (3%)	5 (14%)	7 (18%)	13 (18%)	8 (10%)
Male	30 (79%)	38 (97%)	31 (86%)	31 (82%)	61 (82%)	70 (90%)
**Education:**						
Primary or secondary incomplete	18 (47%)	14 (36%)	31 (86%)	22 (56%)	49 (66%)	36 (46%)
Secondary complete or more	20 (53%)	25 (64%)	5 (14%)	17 (44%)	25 (34%)	42 (54%)†
**Occupation:**						
Subsistence	22 (58%)	25 (64%)	33 (92%)	31 (79%)	55 (74%)	56 (72%)
Professional	16 (42%)	14 (36%)	3 (8%)	8 (21%)	19 (26%)	22 (28%)

RECO – relais communautaires

*P < 0.001.

† *P* < 0.05.

### Characteristics of the children assessed

The characteristics of the children and the mix of presenting conditions were not statistically significantly different between the two groups (**Table 5**). The average age in months was 21.9 for the control group and 20.1 for the intervention group. In regards to the number of children with danger signs, there were 23 (29%) in the intervention group, compared to 14 (19%) in the control group, but the difference was not statistically significant (*P* = 0.129).

**Table 5:** Characteristics of the children and presenting symptoms

	Control (N = 74)	Intervention (N = 78)
	**N (%)**	**N (%)**
**Sex:**		
Male	38 (49)	38 (51)
Female	36 (51)	40 (49)
**Age group (months):**		
<6	12 (16)	9 (12)
6-11	12 (16)	14 (18)
12-23	16 (22)	29 (37)
24-59	34 (46)	26 (33)
**iCCM condition** (can have more than one):		
Fever	71 (96)	71 (91)
Cough/difficulty breathing	44 (59)	51 (65)
Diarrhea	20 (27)	17 (22)
**Presence of danger signs among children with each condition:**		
Fever	11 (15)	20 (28)
Cough/difficulty breathing	3 (7)	12 (24)
Diarrhea	4 (20)	5 (29)
**Complexity of the child’s condition:**		
Single condition	14 (19)	12 (15)
Two conditions	31 (42)	35 (44)
Three conditions or a non-iCCM condition	15 (20)	8 (10)
Danger sign	14 (19)	23 (29)

iCCM **–** integrated community case management

### Quality of care

#### Assessment and Referral for Danger Signs

**Table 6** summarizes the performance of the *RECOs* in identification and referral for danger signs (see **Table 3** for list of danger signs). *RECOs* in the intervention group were more likely to ask about and investigate all relevant danger signs: 63% in the intervention group, compared to 26% in the control group. Controlling for confounders, the *RECOs* in the intervention group were 4.6 more likely to investigate all appropriate danger signs, (aOR = 4.6, 95% CI = 2.1-10.0, *P* < 0.001).

**Table 6:** *RECO* – *relais communautaire* performance on assessment of danger signs and referral

	Correct performance (No. %)		Intervention performance relative to control		
	**Control**	**Intervention**	aOR*	**95% CI**	***P*-value**
**All children**	N = *74*	N = *78*			
All relevant danger signs assessed†	19 (25.7)	49 (62.8)	4.6	(2.1-10.0)	<0.001
Correct referral decision	62 (83.8)	72 (92.3)	4.3	(1.1-16.4)	0.032
**Children with danger signs**	N = *14*	N = *23*			
Correctly referred	7 (50.0)	20 (87.0)	24.2	(1.9-300.2)	0.013
Received correct pre-referral treatment	1 (7.1)	10 (43.5)	68.3‡	(1.6-2813.2)	0.026

*RECO* – *relais communautaire,* aOR – adjusted odds ratio, CI – confidence interval

*Adjusted for age of the child, the condition and complexity of the child’s condition, the age, sex, education and occupation of the *RECO* and the health zone.

†Relevant danger signs here exclude signs obviously absent (lethargy or inability to eat in a child observed breastfeeding) or absent by inference (bloody stools in a child without diarrhea).

‡Adjusted for age of the child, the age, sex, education and occupation of the *RECO* and the health zone.

If a child has no danger sign, the *RECOs* are expected to prompt for symptoms and duration of all three iCCM conditions, regardless of the reason for presenting. The intervention group more consistently performed this step correctly: 95% in the intervention group compared to 74% in the control group. After adjusting for confounders, the *RECOs* in the intervention group were 6.7 times more likely to ask for all three conditions and the duration of any condition present (aOR = 6.7, 95% CI = 1.6-28.0, *P* = 0.009) (**Table 7**). There was no significant difference between the two groups in performance of respiratory count when indicated, although accuracy of the counting was consistently low: 55% in the intervention group and 54% in the control group. Both groups performed well on measuring MUAC. All *RECOs* in both groups performed an RDT when the child presented with fever (not shown).

**Table 7:** *RECO* – *relais communautaire* performance on assessment of sick children for iCCM conditions

	Correct performance (No., %)		Intervention performance relative to control		
	**Control**	**Intervention**	aOR*	**95% CI**	***P*-value**
**All children:**	N = *74*	N = *78*			
Asked for all three conditions	61 (82.4)	74 (94.9)	3.1	(0.8-12.8)	0.103
Asked for all three conditions and duration of each	55 (74.3)	74 (94.9)	6.7	(1.6-28.0)	0.009
**Children 6-59 mo:†**	N = *62*	N = *69*			
Performed MUAC measurement	59 (95.2)	60 (87.0)	0.3	(0.1-1.1)	0.068
MUAC measurement correct	51 (82.3)	58 (84.1)	1.1	(0.4-3.0)	0.868
**Children with respiratory symptoms:**	N = *41*	N = *39*			
Performed breath count at appropriate indication	33 (80.1)	36 (92.3)	2.0‡	(0.4-10.2)	0.32
Breath count correct (within ±3 breaths of evaluator’s count)	22 (53.7)	12 (48.7)	1.0	(0.5-2.2)	0.496

RECO – *relais communautaire,* iCCM – integrated community case management, aOR – adjusted odds ratio, CI – confidence interval, MUAC – mid-upper arm circumference

*Adjusted for age of the child, the condition and complexity of the child’s condition, the age, sex, education and occupation of the *RECO*, the health zone and number of supervisions per month in the health area.

†MUAC (mid-upper arm circumference) is not measured on children under 6 mo

‡Condition and complexity of the child’s condition dropped.

#### Management of individual iCCM conditions

Both groups of *RECOs* performed well on the classification and treatment of fever, correctly classifying 92% of cases in the control group, vs 94% in the intervention group, and providing correct treatment for the child’s age and condition in 63% of cases in the control and 75% in the intervention group (**Table 8**). The odds ratios for both classification and treatment of fever were not statistically significant. In cases of diarrhea, 19% of the control group and 67% of the intervention group provided correct treatment. The small sample size (n = 37) made interpretation of these results difficult, and the odds ratio was not statistically significant (aOR = 5.8, 95% CI = 0.9-38.5, *P* = 0.067). *RECO* performance assessing and treating respiratory conditions was low in both groups: in the control group, 66% correctly classified and 78% correctly treated, while in the intervention group, 67% correctly classified and 67% correctly treated. The odds ratios were not statistically significant. Among cases correctly treated for a respiratory condition in the control group, seven were treated correctly even though they were classified incorrectly.

**Table 8:** *RECO – relais communautaire* performance on classification and treatment of individual iCCM conditions among children without danger signs (N = 115)

	Correct performance (No., %)		Performance relative to control		
	**Control**	**Intervention**	aOR*	**95% CI**	***P*-value**
**Fever/malaria:**	N = *60*	N = *51*			
Correctly classified (RDT done)	55 (91.7)	48 (94.1)	1.2	(0.2-7.2)	0.836
Correctly treated fever with correct dose of ACT and/or paracetamol	38 (63.3)	38 (74.5)	1.7	(0.7-4.1)	0.286
**Diarrhea:**	N = *16*	N = *12*			
Correctly classified	14 (87.5)	12 (100)	n/a		
Correctly treated with correct dose of ORS and zinc	3 (18.8)	8 (66.7)	5.8	(0.9-38.5)	0.067
**Respiratory conditions:**	N = *41*	N = *39*			
Correctly classified after breath count done	27 (65.9)	26 (66.7)	0.7	(0.3-2.3)	0.606
Correctly treated with correct dose of amoxi or comfort measures for cough/cold	32 (78.1)	26 (66.7)	0.6	(0.2-2.0)	0.418

aOR – adjusted odds ratio, CI – confidence interval, iCCM – integrated community case management , RDT – rapid diagnostic test, ORS – oral rehydration salts, n/a – non applicable, RECO – *relais communautaire*

*Adjusted for age of the child, the condition and complexity of the child’s condition, the age, sex, education and occupation of the *RECO* and the health zone.

†Complexity of the child’s condition dropped because of co-linearity.

#### Management of children with any combination of conditions and/or danger signs

Overall correct treatment of children was 39% in the control group and 55% in the intervention group. Adjusting for confounders, *RECOs* in the intervention group were almost three times more likely to provide care consistent with protocol (aOR = 2.9, 95% CI = 1.3-6.3, *P* = 0.010) (**Table 9**). With the addition of a higher standard of correct assessment, the aOR = for the intervention group increased to 3.5 (95% CI = 1.6-8.0, *P* = 0.002). On classification alone, the difference between the two groups was not statistically significant.

**Table 9:** *RECO – relais communautaire* performance on classification and treatment of all children

	Correct performance (No., %)		Difference		
	**Control (N = 74)**	**Intervention (N = 78)**	aOR*	**95% CI**	***P-*value**
Correctly classified	48 *(*64.9)	60 *(*77.0)	2.2	*(*0.9-5.2)	.084
Correctly classified and treated	29 *(*39.2)	43 *(*55.1)	2.9	*(*1.3-6.3)	.010
Correctly assessed, classified and treated	23 *(*31.1)	42 *(*53.9)	3.5	*(*1.6-8.0)	.002

aOR – adjusted odds ratio, CI – confidence interval

*Adjusted for age of the child, the condition and complexity of the child’s condition, the age, sex, education and occupation of the *RECOS* and the health zone.

### RECO workload

On average the assessments and documentation lasted 44.6 minutes (range: 17-81) for the control group and 31.7 minutes (range: 5-59) for the intervention group. Controlling for confounding factors, the *RECOs* in the intervention group spent an average of 10.6 minutes less (95% C = 6.6-14.7, *P* < 0.001) per consultation. The difference was related to time spent on documentation, which took the intervention group on average 0.4 (95% CI = 0.2-0.7) minutes compared to 13.1 minutes for the control group (95% CI = 11.7-14.7, *P* < 0.001).

### Cost analysis

The cost of tools, distribution and training of 100 *RECOs* in one health zone using the MoPH package would be US$ 34 385 compared to US$ 29 967 for the intervention in the first year of roll-out, a savings of US$ 4418. Tool printing costs for the intervention package would be $29 967, $3632 less than the control group. Printing of the laminated job aids, a non-recurrent cost, represents the greatest part of the cost for the intervention package, reducing costs in subsequent years. The control package requires quarterly distribution of tools, while the intervention package requires distribution only twice a year, halving the cost. Training and supervision costs would be unchanged except for the added cost of US$ 490 to print and laminate large format posters called for in the intervention. At the time of the study, the RAcE project implemented iCCM in 11 health zones with approximately 1500 operational *RECOs*. At this scale, the intervention package represents a savings of US$ 66 270 the first year and US$ 90 825 every subsequent year.

### Study limitations

This study has a number of limitations, largely based on the study design. The sample sizes for specific conditions, most notably diarrhea, were small, producing unreliable regression results. The control and intervention areas differed significantly in the age and education of the *RECOs*. These factors were controlled for in the regression analysis to the best of our ability with limited information about the *RECOs*. As mentioned in the methods section, the selection of *RECOs* for the quality of care evaluation was done by zonal project supervisors, and based on availability, possibly resulting in a bias in favor of higher performing *RECOs*, although this would have been equally true for the two arms. The zonal supervisors were not involved in the research design or analysis and the selection process was done independently for the two arms. *RECO* performance may have been influenced by direct observation and performing the assessment at the health facility rather than their usual place of work [13]. While this change in setting may have affected their performance, it also means that the children seen were likely more severely ill than those seen in the village. The role of supervision was not explored in this research, though it would be expected to affect *RECOs* performance [14,15]. The study had no influence on the selection, training, or financial incentives of the supervisors, and was not designed to address supervision. The study design also did not allow differentiation of the effects of the various elements of the intervention package, although this should be included in future studies.

## DISCUSSION

It is well understood that quality of care is critical to achieving positive health outcomes and is determined by a range of issues including provider characteristics, motivation, supervision, the complexity of the guidelines and tasks expected, and trainings and job aids. [16] Our study found that with simplified tools and an adapted training curriculum, a group of low-literate *RECOs* in Tanganyika Province were able to provide higher quality care in less time at lower cost: children treated by *RECO*s in the intervention group were 2.9 times more likely to receive correct treatment; consultations took 10.6 minutes less time per assessment; and cost savings range from US$ 66 000-91 000 per year for a program supporting approximately 1500 *RECOs*.

Overall, the level of performance of the *RECOs* in this study (54% for those in the intervention group) fell within ranges measured in other studies using direct observation and reexamination, ranging from 36% across all three conditions in Burkina Faso [17] to 62% for any uncomplicated condition and 52% for pneumonia alone in Malawi [18] and 64% overall and 72% for pneumonia in Ethiopia [19]. The finding that correct assessment, treatment, and referral, when necessary, of pneumonia cases pose the greatest challenge is consistent with other studies [8,20,21], and has also been found to be true among professional facility based providers [22]. While the results show that the intervention package did not improve quality of care of respiratory conditions, it is possible that the difference in the prevalence of danger signs among those with respiratory conditions (7% in the control vs 24% in the intervention group) skewed the findings. Nonetheless, the low rate of correct treatment overall confirms the importance of developing strategies to improve management of pneumonia.

As in many countries, *RECOs* in the DRC work as volunteers and are not paid for their services, making workload, motivation and retention an important concern [23]. However, there is still not enough evidence globally that payment of community health workers (CHWs) will resolve the issue of motivation and retention and further research on these areas is needed. In interviews conducted as part of another research study [24] with *RECOs* in other health zones in the region, many *RECOs* noted that their work takes up a great deal of time, largely due to the number of tools required to complete, leaving them with limited time for their income-generating activities. Other studies have also found that *RECOs* are frustrated and confused by complex tools and protocols [8] and Guenther et al. [25] have pointed out that complexity also reduces the quality of data and its utility for program improvement. This study found significant time savings with the intervention tool package, 10.6 minutes on average, which when multiplied by the average caseload adds up to over 6 hours per month per *RECO*. In addition to improving quality, simplified tools may improve retention rates by reducing workload and opportunity costs [26].

This research adds important evidence about the potential impact of training and tools on *RECO* performance. Few studies include a control or pre-intervention group [21,27-31]. The only study examining the effect of training or tools looked specifically at the impact of job aids on the correct use of RDTs [32]. Social and gender roles, economic activity, and physical mobility, are all important factors in determining the effectiveness of *RECO*s [33]. Limiting *RECO* selection to individuals who are literate may negatively affect the acceptability and availability of services, as well as retention of *RECO*s. Previous research has found a mixed relationship between characteristics such as literacy, age, and sex and quality of care. Kallander et al [34] found no relationship between these factors and *RECO* performance. In contrast, Crispin et al [35] found that age and educational factors were associated with correct documentation and adherence to protocol, while less literate individuals were equally capable of counseling and enabling their clients. In one study female sex was associated with improved quality of care [36]. The benefits of a training applying adult learning methodologies, improved job aids and simplified tools might be further enhanced if they allowed a relaxation of the selection criteria such that the *RECOs* better reflect the communities they serve.

## CONCLUSION

This study’s findings illustrate that simplifying complex iCCM tools and adapting training curricula to meet the needs of low-literate *RECOs* can result in improved services for children and families in their communities. The elimination and replacement of existing job aids and tools, with pictorial job aids and an integrated register resulted in significant improvement in quality of care and reduced workload. This study also shows that the quality of training can influence the quality of care, an obvious statement, yet one that receives little attention in the research literature. Improvements in training and tools, like those undertaken in the RAcE Project, can be made rapidly and have meaningful impact on health outcomes for children. More attention should be focused on how to maximize *RECO* knowledge and skills using insights from educational approaches designed for adult and low-literacy learners, as part of a larger research agenda on improving CHW performance [16]. Key stakeholders implementing iCCM programs in other countries should ensure that tools are fit for purpose, simplified and adapted to the context and educational levels of CHWs. The results of the study have been accepted by the MoPH and the simplified tools and training curriculum have been adopted at the national level. The tools have also been scaled-up across Tanganyika province. Finally, this research illustrates that even in difficult contexts such as Tanganyika Province in DRC, operational research focused on best practices of iCCM implementation is feasible.
